# Older Adults with Type 2 Diabetes Treated with Metformin: AME-MET Study - A Multicentric Real-world Study in Italy

**DOI:** 10.2174/1871530323666221115091621

**Published:** 2023-04-19

**Authors:** Castellano Elena, Giorgio Borretta, Roberto Attanasio, Boriano Alberto, Daniela Agrimi, Nicola Argese, Cassandra Crescenti, Olga Disoteo, Alessandra Fusco, Enrico Gabellieri, Rinaldo Guglielmi, Giuseppe Lisco, Feliciano Lo Pomo, Maurizio Nizzoli, Annalisa Panico, Barbara Pirali, Antonio Stefano Salcuni, Federica Turchi, Franco Grimaldi

**Affiliations:** 1 Department of Endocrinology, Diabetes and Metabolism, Santa Croce and Carle Hospital, Cuneo, 12100, Italy;; 2 AME Scientific Committee, Milan, 20100, Italy;; 3 Medical Physics Department, Santa Croce and Carle Hospital, Cuneo, 12100, Italy;; 4 District Hospital, Azienda Sanitaria Locale, Brindisi, 72100, Italy;; 5 Department of Endocrinology, S.S. Annunziata Hospital, Taranto, 74100, Italy;; 6 Department of Endocrinology and Diabetes, San Giovanni di Dio Hospital, Florence, 50100, Italy;; 7 Diabetology Department, Niguarda Hospital, Milan, 20100, Italy;; 8 Antidiabetic Center AID, Garibaldi Hospital, Naples, 80100, Italy;; 9 Endocrine and Metabolic Diseases Unit, SS. Antonio e Biagio e Cesare Arrigo Hospital, Alessandria, 15100, Italy;; 10 Department of Endocrinology and Metabolism, Regina Apostolorum Hospital, Albano Laziale, 00041, Italy;; 11 Department of Medicine-Section of Internal Medicine, Geriatrics, Endocrinology and Rare Diseases, Bari, 70100, Italy;; 12 Division of Endocrinology, San Carlo Hospital, Potenza, 85100, Italy;; 13 Endocrinology Unit, Morgagni-Pierantoni Hospital, Forlì, 47121, Italy;; 14 Endocrinology and Diabetology Service, ASL Benevento, 82100, Italy;; 15 Internal Medicine Unit, Humanitas Mater Domini, Castellanza, 21053, Italy;; 16 Endocrinology and Metabolism Unit, University Hospital of Udine, Udine, 33100, Italy;; 17 Endocrinology and Metabolism UOC, INCA-IRCCS, Ancona, 60121, Italy

**Keywords:** Type 2 diabetes mellitus, metformin, dosage, HbA1c level, first-line treatment, anti-diabetic drug

## Abstract

**Aims:**

Metformin is the most widely used drug for the first-line treatment of type 2 diabetes mellitus (T2DM), but its use and schedule have been poorly investigated in elderly patients.

**Methods:**

We conducted an observational, cross-sectional, multicentric study on metformin in T2DM outpatients older than 65 years who were taking the drug for at least 6 months and referred to Italian Endocrinology and Diabetology Services. The primary endpoint was daily metformin dose, and secondary endpoints were the correlations between metformin dose and age, comorbidities, and concomitant use of other drugs. The study was open to all members of AME (Associazione Medici Endocrinologi).

**Results:**

Fifteen Italian centers recruited 751 consecutive participants (42.9% older than 75 years, 48.6% females). T2DM duration was 12.9 ± 9.7 years (longer than 10 years in 53.8%). Metformin had been used for 10.3 ± 6.8 years (longer than 10 years in 52.4%). Metformin dose was 1.6 ± 0.9 g/day (>1.5 g/day in 63.4%). As compared to the youngest, participants older than 75 years did not differ for metformin daily dose or number of administrations. Metformin dose was significantly directly correlated to eGFR, diabetes duration, and metformin treatment duration.

**Conclusion:**

In this real-world study, the minimum daily effective dose of metformin was prescribed in more than half of older T2DM outpatients.

## 1 INTRODUCTION

Type 2 diabetes mellitus (T2DM) is among the most common chronic medical conditions in the elderly population, with an unrelentingly rising prevalence [1, 2]. T2DM significantly increases comorbidity and all-cause mortality, mostly in older adults compared to the general population [3].

Metformin is the most widely used drug worldwide in the treatment of T2DM because it offers an excellent cost-efficacy ratio with a very low risk of hypoglycemia. It can also promote modest weight loss, has long-lasting effects, and good cardio-vascular safety [1, 2, 4].

Clinical guidelines [5, 6] provide specific recommendations for managing patients with T2DM; to date, metformin remains the first-line pharmacologic agent in the absence of contraindications in older adults [7]. It is indicated in patients not reaching their individualized glycemic goals, with or without other anti-diabetic drugs [5].

The use of metformin needs careful consideration in patients with renal failure and aging, as well as other medical conditions potentially increasing the risk of lactic acidosis [8]. Taking into account the age-related physiological decline of renal function, the dose of metformin should be prudently reduced in older adults [7].

According to the last available national data [9], metformin is the most used anti-diabetic drug in Italy in every age group; over 60% of Italian patients with T2DM are indeed treated with metformin alone or in combination with other anti-diabetic medications, including insulin.

The real-world dosage patterns of metformin [10, 11], particularly in elderly patients, have been inadequately investigated. Our study thus evaluated the daily dose of metformin in a large unselected multicentric series of over 65-year-old outpatients with T2DM followed at Endocrinology and Diabetology Services in Italy by taking into account age, comorbidities, and the combination with other anti-diabetic drugs.

## 2 PATIENTS AND METHODS

### 2.1 Design and Protocol

From October 2019, to December, 2020, we performed an observational, cross-sectional, multicenter study involving older adults with T2DM.

Italian Association of Clinical Endocrinologists (Associazione Medici Endocrinologi - AME) commissioned the study that was approved by the Ethical Committee of Cuneo Hospital (ENDO33 n 469/2019) and registered at Clinicaltrials.gov (ID NCT04295031).

Participation in the study was open to all specialists taking care of patients with T2DM in Italy.

The primary endpoint was the evaluation of the daily metformin dose prescribed in outpatient older adults.

Secondary endpoints were possible correlations with age, comorbidities, and the use of other anti-diabetic drugs.

An ad hoc form was developed as an electronic sheet in Excel and used to record all medical findings. The form was emailed to all participating centers that emailed it back to our data manager after filling in the required data.

The following data were required: age, gender, body weight and height, T2DM duration from the diagnosis, metformin treatment duration, metformin schedule (daily dose and number of administrations), use and schedule of other anti-diabetic drugs, and presence of comorbidities requiring drug treatment. Serum creatinine and HbA1c levels obtained within the previous two months were also required.

### 2.2 Patients

Inclusion criteria were T2DM outpatients over 65-year-old, taking metformin (either in the classic formulation or as a modified release tablet) for at least six months, and capable of informed consent.

Exclusion criteria were hospitalization in the previous six months for any cause.

Each center recruited a range of 30-60 participants consecutively.

All patients gave their informed consent, and the Institutional Review Board of each participating center approved the study. All procedures followed were in accordance with the ethical standards of the Helsinki Declaration of 1964, as revised in 2013.

### 2.3 Methods

All biochemical parameters were analyzed in the laboratories of each participating center.

HbA1c was assayed by the ion exchange HPLC method. Serum creatinine levels were assayed by automated analysis using a colorimetric method. The eGFR was calculated for patients under 75-year-old using the following CKD-EPI formula [12]:

eGFR = 141* min (SCr/k, 1) α* max (SCr/k, 1) (-1.209)* and Age (0.993)* [*1.018 if women] [*1.159 if black].

Where SCr represents serum creatinine (in mg/dL), k is 0.7 for women and 0.9 for men, α is −0.329 for women and − 0.411 for men, min represents the minimum of SCr/k or 1, and max represents the maximum of SCr/k or 1.

For a more accurate estimation of kidney function in the elderly, in patients aged 75 or older, the following below-given BIS1 formula [13] was applied:

eGFR= 3736 * Scr^-0.87^ * Age^-0.95^[* 0.82 if female].

For statistical purposes, we evaluated the eGFR in patients aged 75 or older using the CKD-EPI formula. Body mass index (BMI) was calculated as the body weight in kilograms divided by the squared body height in meters. Insulin, sulfonylureas and glinides were considered drugs with increased hypoglycaemic risk.

### 2.4 Statistical Analysis

Variables were preliminarily tested for normal distribution with the Shapiro-Wilk’s W test, and data were expressed as mean ± standard deviation (SD) when normally distributed or as the median and interquartile range (IQR) when not normally distributed. Continuous variables with non-normal and normal distribution were analyzed by Mann-Whitney U test and t-test for unpaired samples, respectively, as appropriate. Differences in categorical variables were analyzed by χ^2^ or Fisher’s test, as appropriate. Linear regression analysis was used to evaluate the correlations between the metformin dose and the other continuous variables. Variables that were significant in univariate analysis were entered into multiple regression analysis.

The level of statistical significance was set at p ≤0.05. The calculations were performed using SPSS (IBM SPSS Statistics – Version 21).

## 3 RESULTS

The study was performed in 15 Italian centers, homogenously distributed throughout Italy.

We enrolled 751 participants, 51.4% males and 48.6% females, aged 74.1 ± 6.4 years (42.9% ≥75-yo).

Table **1** summarizes the demographic, clinical and biochemical data of the whole study group.

**Abbreviations:** BMI = Body Mass Index, HbA1c= Glycaated Hemoglobin, eGFR= Estimated Glomerular Filtration Rate.

Patients were meanly overweight, with fair glycemic control. The renal function was generally preserved; only 7.3% had eGFR <45 mL/min/1.73 m^2^. Diabetes duration was longer than 10 years in more than half of the participants, with a mean of 12.9 ± 9.7 years. Metformin therapy had been administered for at least 10 years in more than half of the patients, and only 28.8% had been treated for five years or less. Moreover, anti-diabetic drugs with increased hypoglycemic risk were ongoing in 37.2% of patients (insulin in 20.4%). The mean number of co-morbidities requiring pharmacological treatment was 2.4, greater than 4 in about a quarter of participants.

Table **2** shows the comparison between patients subdivided according to age (under or over 75), daily metformin dose (under or over 1.5 g) or concomitant use of anti-diabetic drugs with increased risk of hypoglycemia.

Briefly, patients over 75-year-old had significantly lower BMI, lower eGFR, longer disease duration and metformin use duration than the younger patients. Patients treated with a daily dose of metformin >1.5 g had higher eGFR, longer diabetes duration and metformin use duration. Patients on treatment with drugs at higher potential risk of hypoglycemia had worst glycemic control, longer diabetes duration and metformin use duration than the remaining. In addition, they were treated with a higher number of daily metformin administrations.

Table **3** shows univariate analysis; daily metformin dose was significantly directly correlated with eGFR, diabetes duration, and metformin treatment duration.

Considering the diabetes duration and the metformin treatment duration highly related (ß = 0.807, p < 0.001, VIF > 3), the diabetes duration was then excluded by the multiple regression model. In the multivariate regression analysis, daily metformin dose remained significantly associated with eGFR (ß = 0.066, p = 0.029) and with the duration of metformin treatment (ß = 0.129, p = 0.001).

The correlations were also tested by applying the CKD-EPI formula to the whole series regardless of age; all the statistically significant relationships were confirmed (data not shown).

## 4 DISCUSSION

This observational cross-sectional multicentric study, including a large series of older adult outpatients with long-lasting T2DM, shows that daily metformin dose was equal to or greater than 1.5 g in most cases, regardless of age. Moreover, the mean metformin daily dose was higher in patients treated concomitantly with anti-diabetic drugs with hypoglycemic risk and was closely related to renal function.

To date, treatment guidelines from most scientific societies [5, 6] recommend metformin as a first-line pharmacologic treatment for T2DM, mainly due to its glycemic benefit with a relatively safe profile of adverse effects, particularly with the avoidance of hypoglycemia. Moreover, other potential beneficial roles of metformin were reported on organs typically affected by diabetes complications, as well as cancer risk reduction and improvement in cognitive function [14].

The most common side effect of metformin is gastrointestinal discomfort, whose impact is lower if the drug is titrated gradually [15]. However, lactic acidosis, even though rarely reported during metformin use, warrants caution in patients with renal failure and advanced age [8].

Taking into account the physiological decline of renal function in older adults, a cautious reduction of metformin dose is strongly recommended [7]. Depending on renal function, clinical guidelines advise avoiding the use of metformin or lowering doses [7, 17].

Our study shows that in older adults, metformin dose is positively correlated to renal function, with eGFR being an independent predictor of daily metformin dose. Notably, only a small minority of older adults treated with metformin had CKD stage G3b (and none a higher stage).

It can therefore be assumed that Italian diabetologists tend to adopt a prudential approach in older adults, probably aimed at avoiding risks related to possible further decline in renal function. This cautious approach could be related to the integrated disease management model, common in many Italian regions, with the general practitioners taking care of the short to medium-term follow-up of T2DM patients [18]. On the other hand, the availability of alternative therapies without hypoglycemic risk and with demonstrated safety in patients with renal insufficiency, whose cost is reimbursed by the Italian National Health Service, might have played a key role in the therapeutic approach. Metformin is widely prescribed worldwide for the treatment of T2DM [19]; similarly, according to the last available national data [9, 20], it is the most used anti-diabetic drug in Italy in every age group, alone or in combination with other anti-diabetic medications, including insulin.

The efficacy of metformin is dose-dependent [19, 21], with maximal benefits observed at the upper limits of the recommended daily dosage [21-23] and a minimum fully effective dose of 1.5 g/day [22]. Nevertheless, the real-world dosage patterns of metformin have been so far poorly investigated [10, 11], particularly in the elderly. The available real-world studies on metformin dosage patterns in the United States and the United Kingdom focused mainly on the first months of treatment [10] and its use in monotherapy [11]. They reported a suboptimal dosage of the drug. Mahabaleshwarkar and DeSantis [10] reported that patients older than 65 years had lower odds of receiving 1.5 g/day of metformin at initiation than younger patients. The daily dose of metformin was equal to or greater than 1.5 g in most older adults in this series. Moreover, daily metformin dose was higher in patients on concomitant treatment with other anti-diabetic drugs at potential risk of hypoglycemia, which was the same subset of patients with longer duration of T2DM and worst glycemic control. Italian diabetologists seem to be particularly careful about renal function decline but, at the same time, use effective doses of metformin in patients with longer duration of T2DM.

It is worth noting that metformin had been administered for long periods in the older adults included in our study; the average was longer than ten years. Therefore, our observation reflects a period following any late titration. This is a relevant difference compared to previous real-world studies [10, 11], whose results might not be representative of the steady state after the titration period, particularly in the elderly.

Along the same line, the finding in our series of a significantly higher prevalence of gastrointestinal side effects in patients taking lower daily doses of metformin supports the hypothesis that titration had been completed at the moment of the evaluation.

Regarding metformin treatment maintenance in older adults, recommendations about stopping the use of metformin in older people with T2DM were published. A systematic review highlighted the lack of good-quality evidence on the risks and benefits of metformin for the management of T2DM in older people [17]. The use of metformin seems associated with lower mortality risk in older people, and with a reduced risk of adverse events, such as hypoglycemia and nonfatal cardiovascular events, in comparison to other anti-diabetic drugs, especially sulfonylureas. However, no prospective studies focusing on very old and functionally and cognitively impaired people are available yet.

A retrospective observational administrative database study in a primary care setting performed in an Italian region [24] evaluated the utilization patterns of anti-diabetic drugs at initiation and over 1 year of follow-up. It was reported that metformin was the most prescribed drug, both as monotherapy and as a fixed combination with other drugs, even in patients over 80 years. Most patients persisted on metformin at 12 months after initiation.

The strengths of this study are inherent in its design, reflecting the current therapeutic approach to T2DM in real-life practice. To the best of our knowledge, this is the first study exploring metformin treatment modalities in real life, specifically in older adults.

As for the limitations, this study focused on a homogeneous population, in terms of ethnicity and lifestyle, from various regions in Italy. Therefore, these findings cannot be generalized to patients with T2DM from other countries and ethnic groups with different health system organizations. Even though the patients were consecutively recruited, the representativeness of participating patients and centers could not be proven. A significant proportion of T2DM patients treated with metformin are followed only by general practitioners in Italy. More than 20% of patients in this study were on concomitant treatment with metformin and insulin, indicating the likely selection of the more severe cases of diabetes.

However, no data are available on islet function that can affect the prescription of metformin. The C-peptide and insulin evaluation are not routinely performed during routine outpatient visits but are required only in selected cases. Further prospective studies could give detailed insight into this topic.

It must be considered that eGFR determination may be affected by multiple other variables beyond diabetes and albuminuria [25], which are not discussed in our series. This eGFR value is routinely provided by our laboratories, reflecting what is taken into account by Italian Diabetologists in their current clinical practice.

Finally, it is to note that our study was conducted at a time when the latest guidelines had just been published and were still poorly applied in real clinical practice, with an under-prescription of innovative anti-diabetic drugs in Italy, particularly in older adults [20].

## CONCLUSION

In conclusion, in Italy, the metformin dosage pattern in older adults with T2DM is based on efficacy criteria, with the minimum daily effective dose prescribed in more than half of patients. At the same time, the therapeutic approach appears to be cautious, particularly regarding the decline of renal function. The generalizability of our results is limited as healthcare systems, reimbursement policies, and access to different treatment options are country-specific. In this regard, further research should focus on comparison among countries.

## Figures and Tables

**Table 1:** Demographic, biochemical and clinical features of the whole series (n = 751).

**Age** (years) 65-74 75-84 ≥85	74.1 ± 6.4 57.1% 36.4% 6.5%
**Male sex**	51.4%
**BMI** (kg/m^2^)	29.4 ± 5.3
**HbA1c** (%)	7.2 ± 3.7
(mmol/mol)	55 ± 17
**eGFR** (mL/min/1.73 m^2^) >60 45-60 <45 <30	65.2 ± 9.3 51.8% 40.9% 7.3% 0%
**Diabetes duration** (years) ≤5 5.1-10 >10	12.9 ± 9.7 20.1% 26.1% 53.8%
**Metformin use** (years) ≤5 5.1-10 >10	10.3 ± 6.8 28.8% 18.8% 52.4%
**Metformin daily dose** (g/day) <1.5 ≥1.5	1.6 ± 0.9 36.6% 63.4%
**Metformin administrations** (N/day)	2 ± 0.7
**Gastrointestinal side effects**	11.2%
**Metformin modified release**	22%
**Metformin alone**	14.3%
**Diabetes therapies with hypoglycemic risk**	37.2%
**Insulin use**	20.4%
**Comorbidities requiring pharmacologic treatment** ≤2 3–4 >4	2.4 ± 1.3 34% 39.4% 26.6%

**Note:** Data are expressed as mean ± SD or as percentage as appropriate.

**Table 2:** Comparison between results of patients subdivided according to age, metformin daily dose, and concomitant use of other antidiabetic drugs with risk of hypoglycaemia.

**-**	**Age (Years)**		***p***	**Metformin Dose (g/day)**		***p***	**On treatment with Increased Risk of ** **Hypoglycemia**		***P***
-	<75	≥75		<1.5	≥1.5	-	Not	Yes	-
N	57.1%	42.9%		36.6%	63.4%	-	62.8%	36.6%	-
Age (years)	-	-		73.7±6.2	74.6 ± 6.6	0.062	73.9±6.4	74.6±6.4	0.239
BMI (kg/m^2^)	30.1±5.6	28.4±4.6	<0.001	29.5±5.3	29.3±5.2	0.69	29.3±5.2	29.6±5.4	0.386
HbA1c (%) (mmol/mol)	7.1±2.9 54±8	7.4±3.8 57±18	0.339	7.3±0.5 56±21	7.2±1 55±3	0.078	6.9±2.7 52±6	7.9±0.5 63±21	0.001
eGFR (mL/min/1.73 m^2^)	69.3±13.9	55.9±12.3	<0.001	62.1±15.2	66.5±14.1	<0.001	64.7±14.9	63.5±14.6	0.340
Diabetes duration (years)	11.8±10.2	14.4±8.5	<0.001	11.3±10	14.7±8.6	<0.001	10.5±7	17.9±12.3	<0.001
Metformin use (years)	9.6±6.7	11.4±6.9	0.001	8.7±6.3	11.8±7	<0.001	8.8±6.4	13.2±6.9	<0.001
Metformin daily dose (g/day)	1.64±0.7	1.61±1	0.653	-	-		1.5±0.6	1.8±1.3	<0.001
Metformin administrations (N/day)	2.1±0.7	2±0.7	0.572	1.8±0.7	2.3±0.5	<0.001	2±0.7	2.2±0.7	<0.001
Gastrointestinal side effects	11.1%	11.2%	0.514	16.6%	5.1%	<0.001	12.5%	7.4%	0.039
Insulin use	20.7%	18.1%	0.236	16.1%	24.9%	0.002	0	60%	
Number of comorbidities	2.3±1.3	2.5±1.3	0.250	2.41±1.3	2.45±1.2	0.114	2.4±1.3	2.5±1.2	0.291

**Note:** Data are expressed as mean ± SD or as percentage as appropriate. **Abbreviations:** BMI = Body Mass Index, HbA1c= Glycaated Hemoglobin, eGFR= Estimated Glomerular Filtration Rate.

**Table 3:** Univariate analysis with metformin dose as the independent variable.

**-**	**Beta Value**	**P Value**
Age	-0.015	0.69
BMI (kg/m^2^)	0.001	0.972
HbA1c (%)	0.004	0.662
eGFR (mL/min/1.73 m^2^)	0.146	<0.001
Diabetes duration (years)	0.11	0.002
Metformin use (years)	0.143	< 0.001
Number of comorbidities	-0.052	0.072

Moreover, guidelines confirm metformin as the first-line treatment for T2DM in older adults [7], which are typically considered a fragile population due to their increased frequency of comorbidities [16].

## Data Availability

The data that support the findings of this study are available upon request from the corresponding author.

## LIST OF ABBREVIATIONS

AME: Associazione Medici Endocrinologi (Italian Association of Clinical Endocrinologists)
BMI: Body Mass Index
CKD-EPI: Chronic Kidney Disease Epidemiology Collaboration
eGFR: Estimated Glomerular Filtration Rate
HbA1c: Glycated hemoglobin
HPLC: High-Pressure Liquid Chromatography
IQR: Interquartile Range
SD: Standard Deviation
T2DM: Type 2 Diabetes Mellitus
